# Supplementary material for: Long term carbon export from mountain forests driven by hydroclimate and extreme event driven landsliding
Source: Commun Earth Environ. 2025 Jun 4;6(1):432. doi: 10.1038/s43247-025-02382-2 (PMC12137134; doi:10.1038/s43247-025-02382-2)
Supplement: Supplementary file 2 — Supplementary Material [file 43247_2025_2382_MOESM2_ESM.pdf]

Supplementary information: Long term carbon export from mountain forests driven by hydroclimate and extreme event driven landsliding

Jamie D. Howarth[1,8], Sean J. Fitzsimons[2], Adelaine Moody[1], Jin Wang[3,4], Mark Garnett[5], Thomas Croissant[3], Alex L. Densmore[3], Andy Howell[6,7] and Robert G. Hilton[8]

[1]Victoria University of Wellington, Wellington, New Zealand.

[2]University of Otago, Dunedin, New Zealand.

[3]Durham University, Durham, UK

[4] State Key Laboratory of Loess and Quaternary Geology, Institute of Earth Environment, Chinese Academy of Sciences, Xi'an, China

[5]NERC Radiocarbon Facility, East Kilbride, UK

[6]GNS Science, Lower Hutt, New Zealand

[7]University of Canterbury, Christchurch, New Zealand

[8]University of Oxford, Oxford, UK

Corresponding author: Jamie Howarth (Jamie.Howarth@vuw.ac.nz)

Contents:

Supplemental Figures:

Supplementary Figure 1: Maps of lakes Paringa and Mapourika showing the lake bathymetry and location of the cores used to reconstruct sedimentary fill volumes.

Supplementary Figure 2: Example of intra-lake correlations used to model the volumes of the sedimentary fill for Lake Paringa.

Supplementary Figure 3: Example of intra-lake correlations used to model the volumes of the sedimentary fill for Lake Mapourika.

Supplementary Figure 4: Thickness grids for each co-seismic, post-seismic, inter-seismic phase for which volumes were modelled for Lake Paringa.

Supplementary Figure 5: Thickness grids for each co-seismic, post-seismic, inter-seismic and storm-induced landsliding phase for which volumes were modelled for Lake Mapourika.

Supplementary Figure 6: Age-depth models for the Lake Paringa (a) and Lake Mapourika (b) master cores.

Supplementary Figure 7: Mixing model between bedrock (OC_{petro}) and biospheric (OC_{bio}) organic carbon end members based on $F^{14}C$ and $\delta^{13}C$.

Supplementary Figure 8: The relationship between the fraction of OC that is OC_{petro} (F_{petro}) and $\delta^{13}C$.

Supplementary Figure 9: Comparison of suspended sediment (SS) and organic carbon (OC) yields derived from the lake based reconstructions to equivalent yields from global rivers.

Supplementary Data:

Supplementary Data 1: Volumes, densities, water contents and sediment and carbon masses for lakes Paringa and Mapourika.

Supplementary Data 2: Suspended sediment, OC_{bio} and OC_{petro} yields for lakes Paringa and Mapourika.

Supplementary Data 3: Organic geochemistry measurements for Lake Mapourika.

Supplementary Data 4: ^{137}Cs measurements for master cores from lakes Paringa and Mapourika.

Supplementary Videos:

Supplementary Video 1: Movie of reconstructed sedimentary fill volume model for Lake Paringa

Supplementary Video 2: Movie of reconstructed sedimentary fill volume model for Lake Mapourika

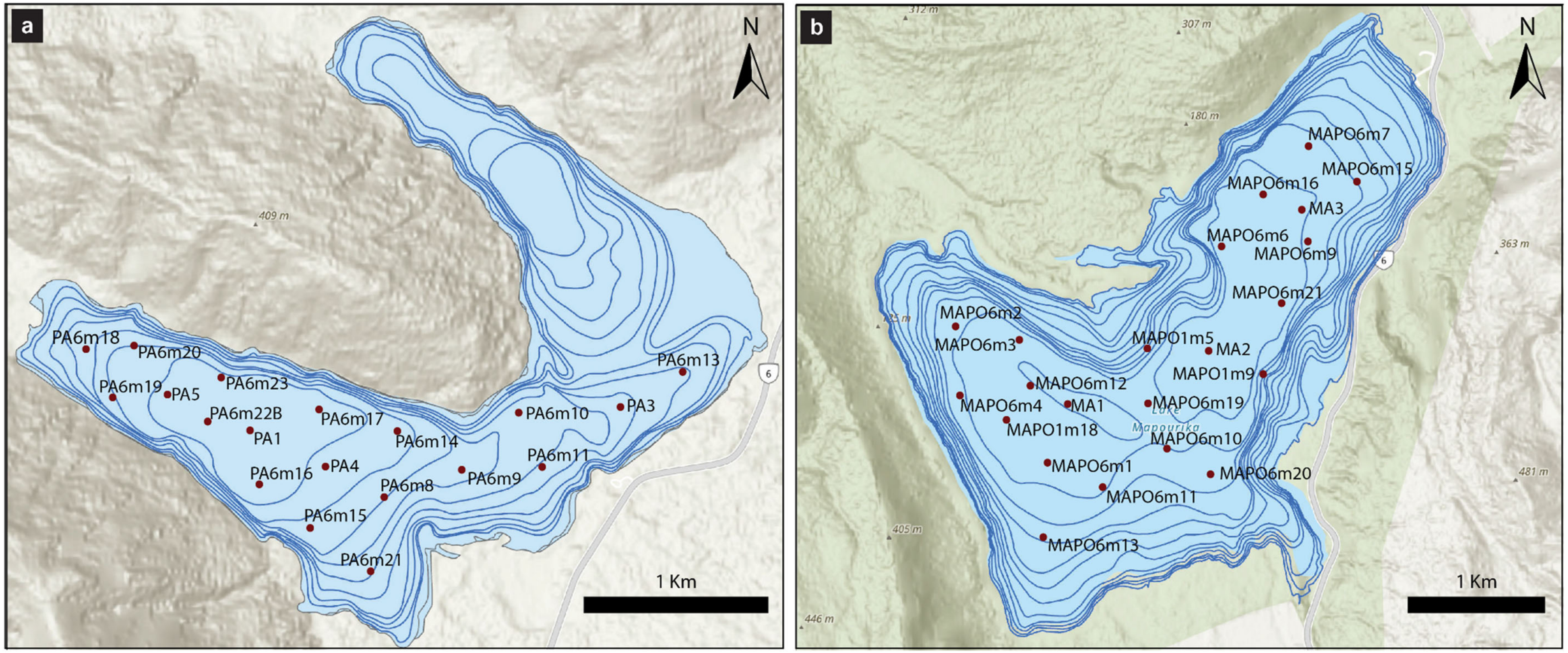

Supplementary Figure 1: Maps of lakes Paringa (a) and Mapourika (b) showing the bathymetry of the lakes and the location of the cores used to reconstruct sedimentary fill volumes.

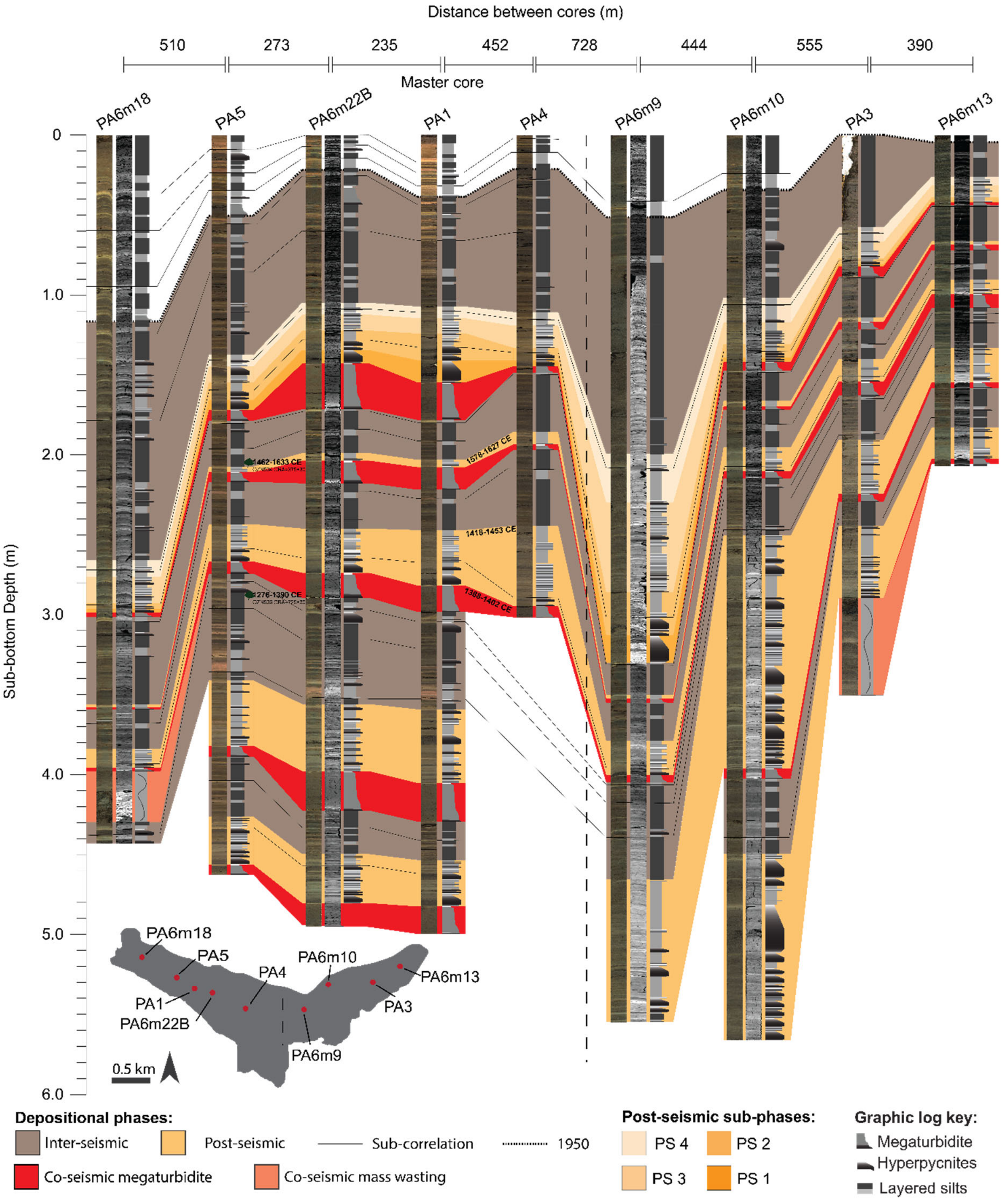

Distance between cores (m)
510
273
235
452
728
444
555
390
Master core
PA6m18
PA5
PA6m22B
PA1
PA4
PA6m9
PA6m10
PA3
PA6m13
Sub-bottom Depth (m)
0
1.0
2.0
3.0
4.0
5.0
6.0
1462-1633 CE
1578-1827 CE
1418-1453 CE
1276-1390 CE
1388-1402 CE
PA6m18
PA5
PA4
PA6m10
PA6m13
PA1
PA6m22B
PA3
PA6m9
0.5 km
Depositional phases:
Inter-seismic
Post-seismic
Sub-correlation
1950
Co-seismic megaturbidite
Co-seismic mass wasting
Post-seismic sub-phases:
PS 4
PS 2
PS 3
PS 1
Graphic log key:
Megaturbidite
Hyperpycnites
Layered silts

Supplementary Figure 2: Example of intra-lake correlations used to model the volumes of the sedimentary fill in Lake Paringa. Correlations are derived from distinct lithofacies stacking patterns visual in optical images, visual logs and CT density of the cores and demonstrate that co-, post- and inter-seismic units can be correlated throughout Lake Paringa's Windbag Basin. These basin wide correlations are supported by sub-correlations that can be traced between adjacent cores and validated by radiocarbon dates that are consistent with the age of the correlations established using the age-depth model for the master core. See data repository for correlations between the remaining cores (https://figshare.com/projects/Long_term_carbon_export_from_mountain_forests_driven_by_hydroclimate_and_extreme_event_driven_landsliding/125050).

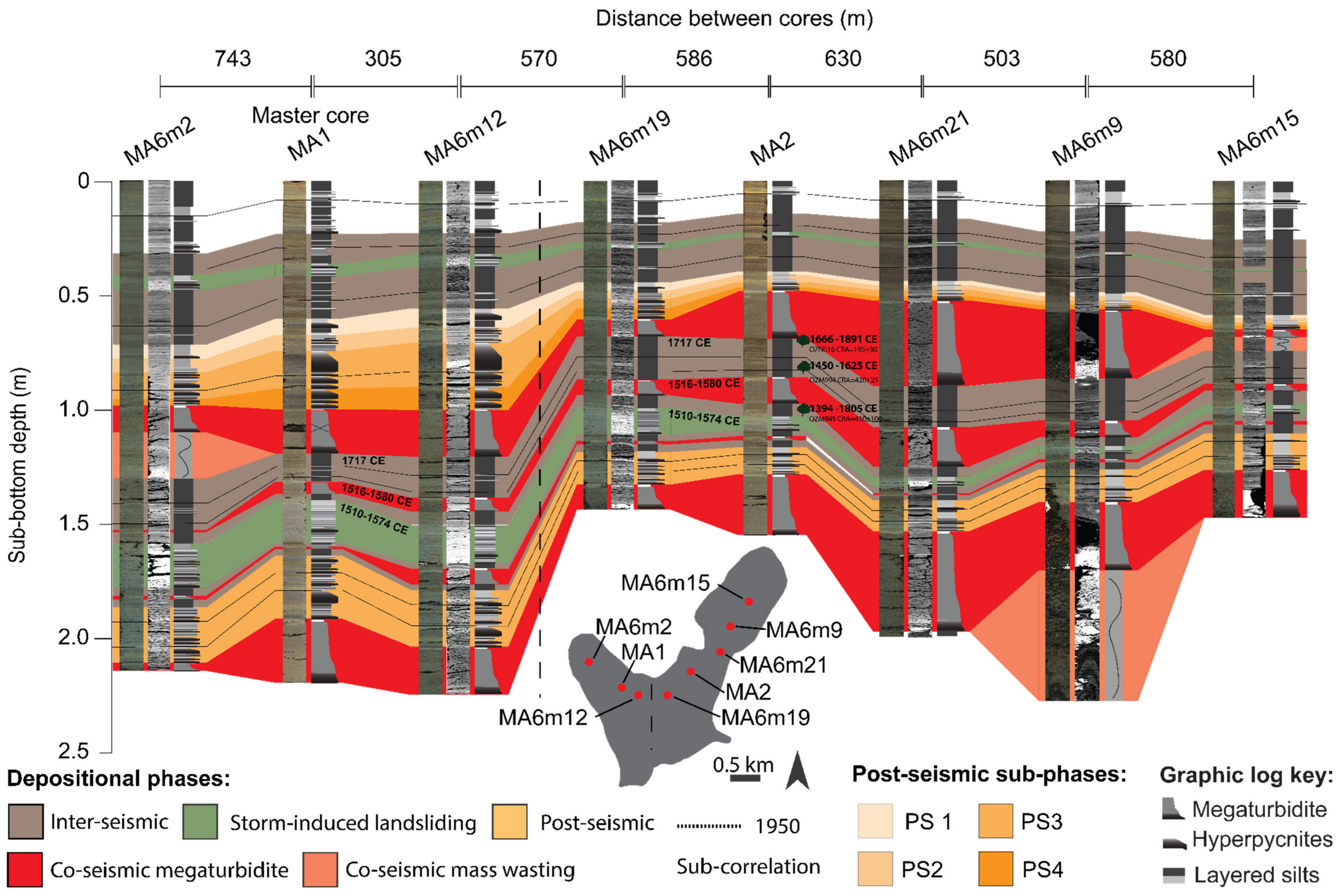

Distance between cores (m)
743
305
570
586
630
503
580
Master core
MA6m2
MA1
MA6m12
MA6m19
MA2
MA6m21
MA6m9
MA6m15
Sub-bottom depth (m)
0
0.5
1.0
1.5
2.0
2.5
1717 CE
1516-1580 CE
1510-1574 CE
1666-1891 CE
1450-1625 CE
1394-1805 CE
0.5 km
Depositional phases:
Inter-seismic
Storm-induced landsliding
Post-seismic
1950
Sub-correlation
Co-seismic megaturbidite
Co-seismic mass wasting
Post-seismic sub-phases:
PS 1
PS2
PS3
PS4
Graphic log key:
Megaturbidite
Hyperpycnites
Layered silts

Supplementary Figure 3: Example of intralake correlations used to model the volumes of the sedimentary fill in Lake Mapourika. Correlations are derived from distinct lithofacies stacking patterns visual in optical images, visual logs and CT density of the cores and demonstrate that co-, post- and inter-seismic units can be correlated throughout Lake Mapourika. These basin wide correlations are supported by sub-correlations that can be traced between adjacent cores and validated by radiocarbon dates that are consistent with the age of the correlations established using the age-depth model for the master core. See data repository for correlations between the remaining cores (https://figshare.com/projects/Long_term_carbon_export_from_mountain_forests_driven_by_hydroclimate_and_extreme_event_driven_landsliding/125050).

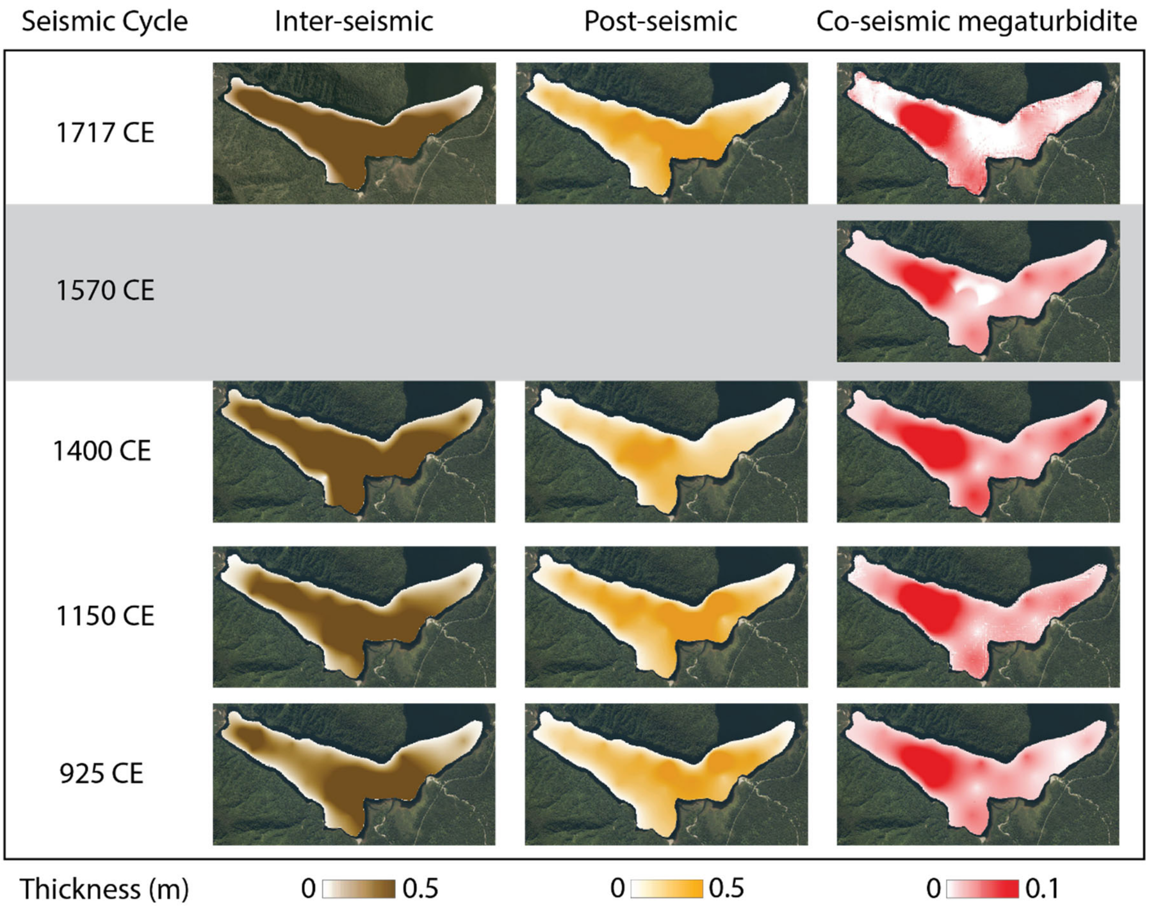

Supplementary Figure 4: Thickness grids for each co-seismic, post-seismic, inter-seismic phase for which volumes were modelled for Lake Paringa. There is a sedimentary signature of seismic shaking and related minor increase in suspended sediment yield at 1570 CE likely associated with rupture of a fault within the Southern Alps[1,2]. As this earthquake was not associated with the Alpine Fault we include the post-seismic (which is minor) and the inter-seismic accumulation in the inter-seismic phase for the 1400 CE earthquake cycle that extends from the end of the 1400 CE earthquake post-seismic phase until the 1717 CE earthquake.

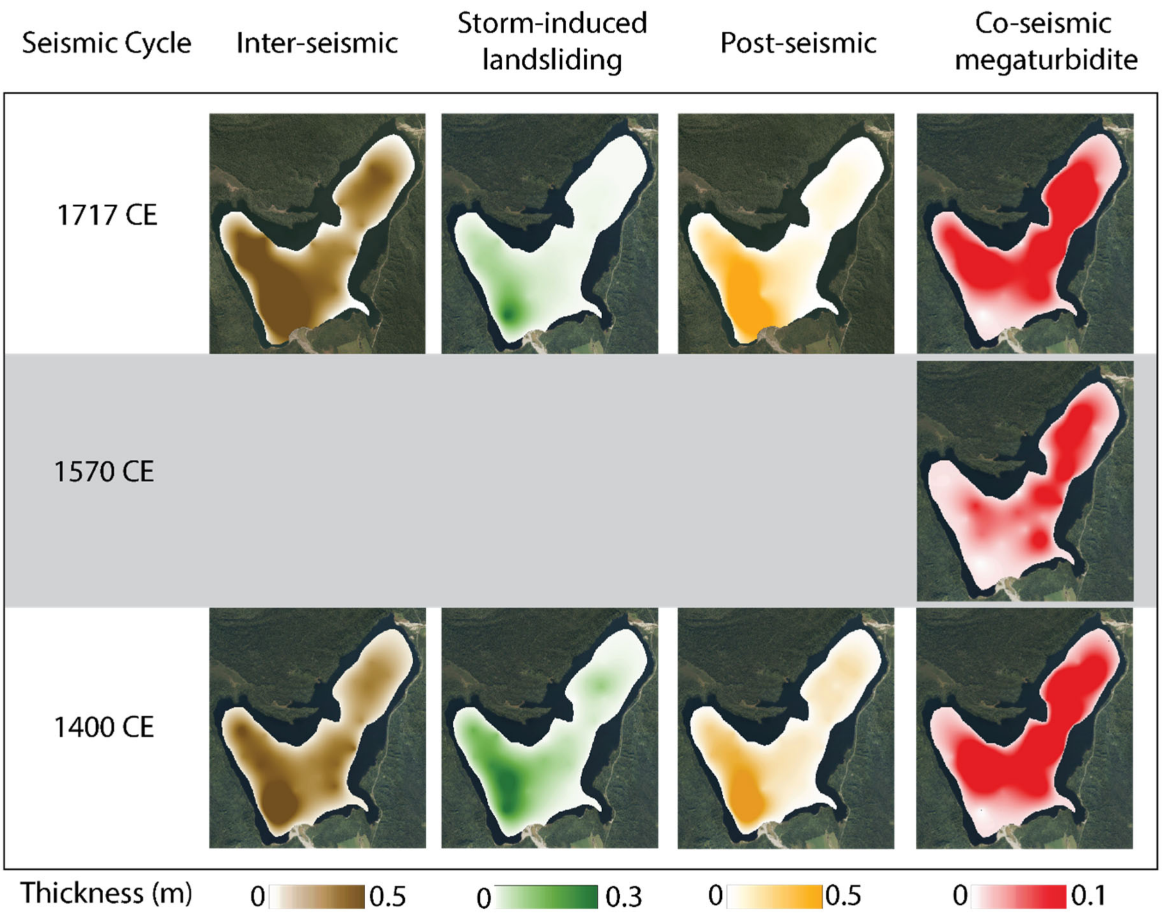

Supplementary Figure 5: Thickness grids for each co-seismic, post-seismic, inter-seismic and storm-induced landsliding phase for which volumes were modelled for Lake Mapourika. There is a sedimentary signature of seismic shaking at 1570 CE likely associated with rupture of a fault within the Southern Alps[1,2] but unlike Lake Paringa no increase in suspended sediment yields was observed.

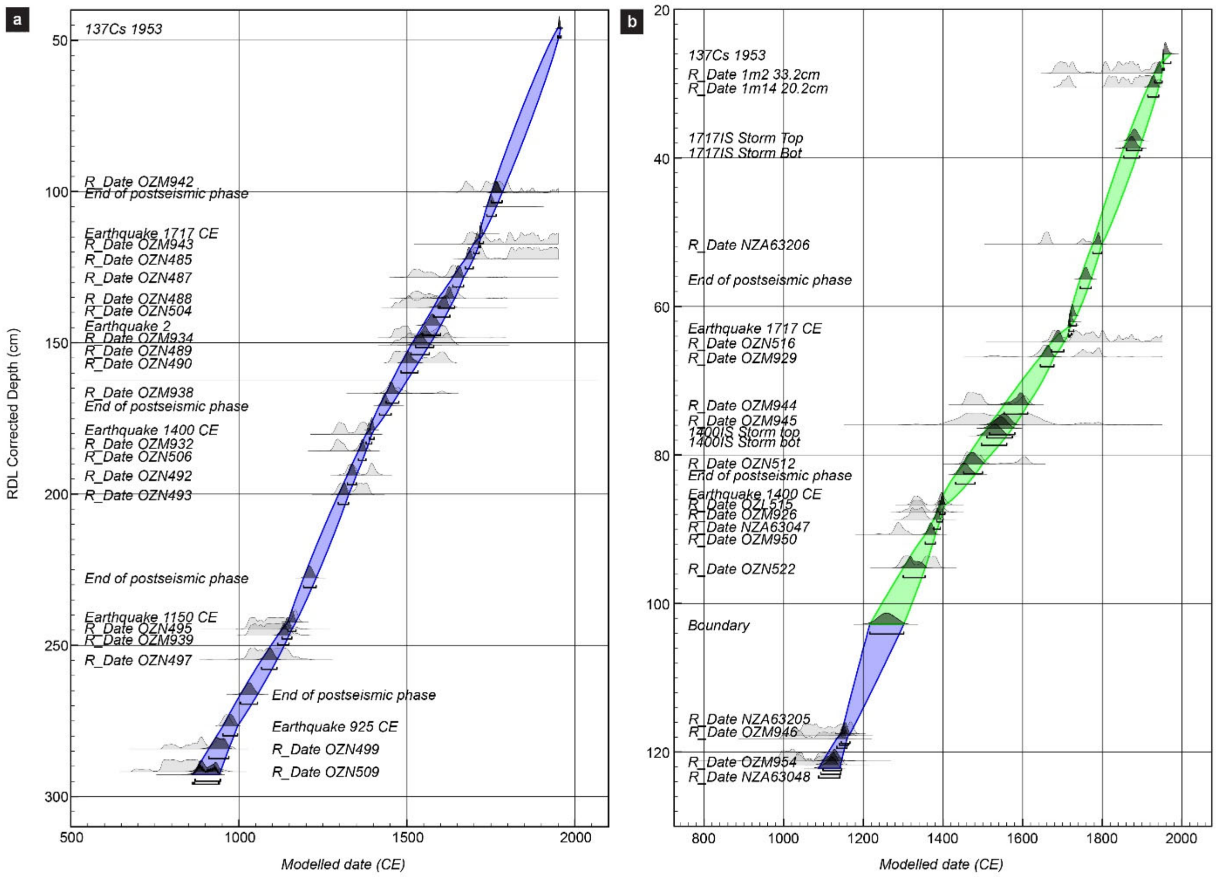

Supplementary Figure 6: Age-depth models for the Lake Paringa (a) and Lake Mapourika (b) master cores (PA1 and MA1, respectively). Chronology was derived from ^{137}Cs, ^{14}C dates and previously defined earthquake ages from ref.[3]. Radiocarbon dates were calibrated with the SHCal20 calibration curve[4] and integrated with the other chronology using the P_Sequence algorithm in OxCal 4.4 (ref.[5]). Curves for each sample show the calendar age likelihood (light grey) and posterior probability density functions (PDFs) (dark grey) for all sources of chronology, as well as the age model uncertainty at the 95% level of confidence (blue and green regions).

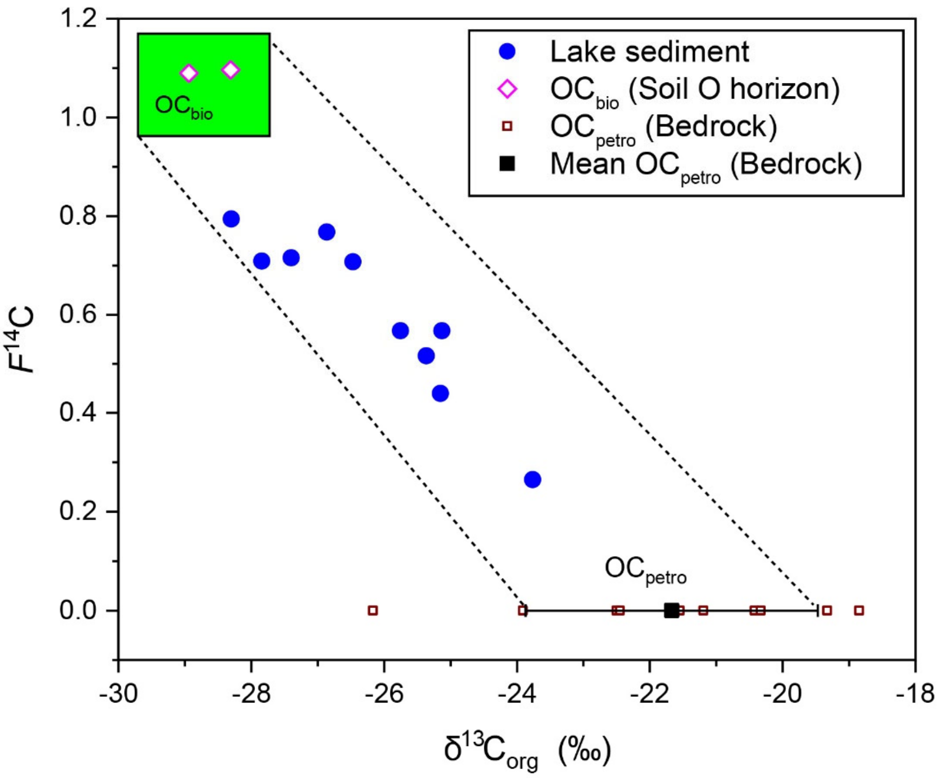

Supplementary Figure 7: Mixing model between bedrock (OC_{petro}) and biospheric (OC_{bio}) organic carbon end members based on $F^{14}C$ and $\delta^{13}C$. The model shows that Lake Mapourika samples are an admixture of both forms of OC. OC_{petro} endmember samples are from Hilton et al.[6], while OC_{bio} endmember samples (in the green box) are from Wang et al.[7].

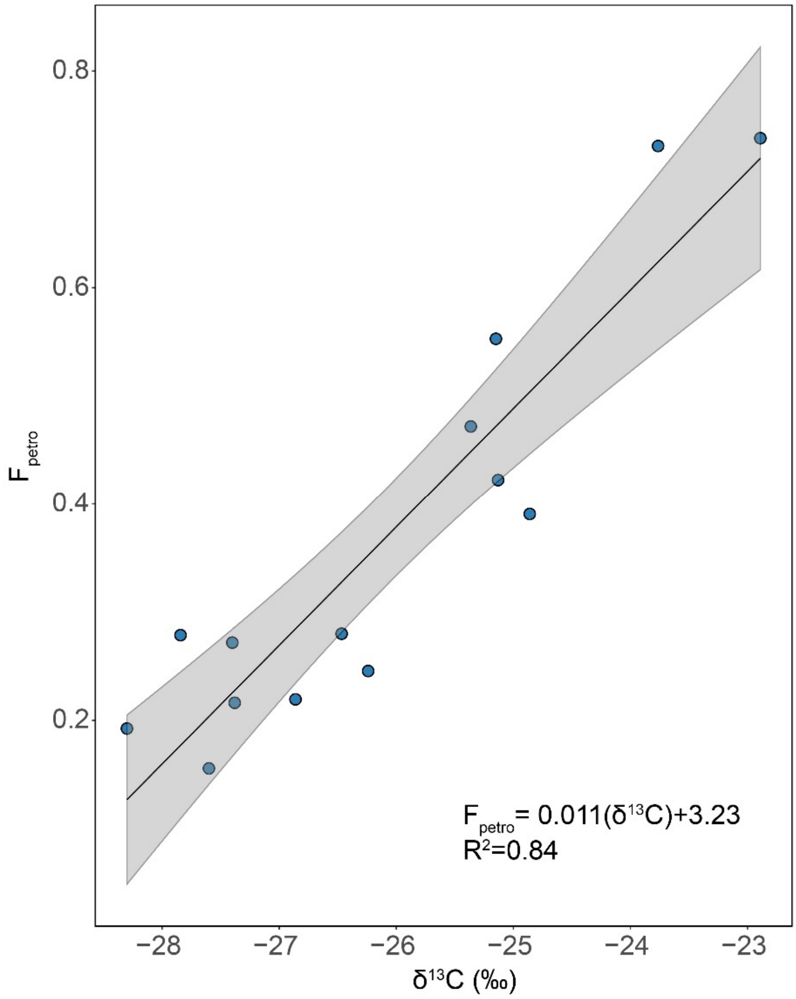

Supplementary Figure 8: The relationship between the fraction of OC that is OC_{petro} (F_{petro}) and δ^{13}C. A linear regression analysis shows that there is a significant linear relationship between δ^{13}C and F_{petro}, such that 84% of the variance in F_{petro} can be explained by variation in δ^{13}C. As the number of δ^{14}C measurements are more limited we use δ^{13}C and the linear equation produced by the regression to predict F_{petro} for each phase the Lake Mapourika master core. Hence, the OC in each phase can be parsed into OC_{petro} and OC_{bio} fractions.

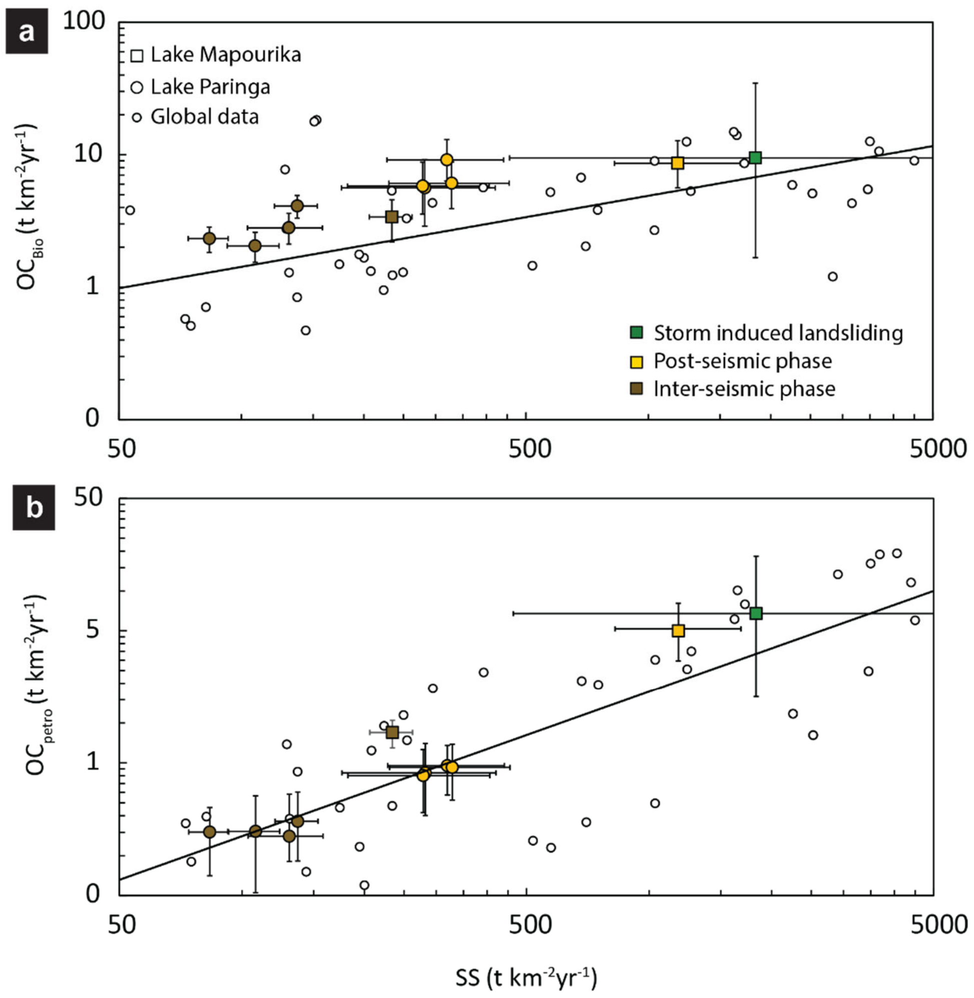

Supplementary Figure 9: Comparison of suspended sediment (SS) and organic carbon (OC) yields derived from the lake-based reconstructions to equivalent yields from global rivers. The relationship between SS and OC_{bio} yields (a), and SS and OC_{petro} yields (b) for post-seismic, inter-seismic and storm-induced landsliding phases. SS and OC_{bio} yields reconstructed from the lakes are compared to the global dataset derived from gauging of rivers from Hilton and West[8]. Error bars show 95% Highest Probability Density Function ranges. Equation for trend line in a. $OC_{bio}= 0.1207SS^{0.5394}$; $R^2 = 0.48$; P value = <0.001. Equation for trend line in b. $OC_{petro}= 0.0.0009SS^{1.09123}$; $R^2 = 0.93$, P value = <0.001).

Supplementary Data:

Supplementary Data 1: Volumes, density, water content and sediment and carbon masses for lakes Paringa and Mapourika.

(https://figshare.com/projects/Long_term_carbon_export_from_mountain_forests_driven_by_hydroclimate_and_extreme_event_driven_landsliding/125050).

Supplementary Data 2: Suspended sediment, OC_{bio} and OC_{petro} yields for lakes Paringa and Mapourika.

(https://figshare.com/projects/Long_term_carbon_export_from_mountain_forests_driven_by_hydroclimate_and_extreme_event_driven_landsliding/125050).

Supplementary Data 3: Organic geochemistry measurements for Lake Mapourika.

(https://figshare.com/projects/Long_term_carbon_export_from_mountain_forests_driven_by_hydroclimate_and_extreme_event_driven_landsliding/125050).

Supplementary Data 4: ^{137}Cs measurements for master cores from lakes Paringa and Mapourika.

(https://figshare.com/projects/Long_term_carbon_export_from_mountain_forests_driven_by_hydroclimate_and_extreme_event_driven_landsliding/125050).

Supplementary Videos

Volume model for Lake Paringa

Volume model for Lake Mapourika

Supplementary References

1 Briggs, J., Robinson, T. & Davies, T. Investigating the source of the c. AD 1620 West Coast earthquake: implications for seismic hazards. *New Zealand Journal of Geology and Geophysics* **61**, 376-388 (2018). https://doi.org:10.1080/00288306.2018.1499537

2 Howarth, J. D. *et al.* Past large earthquakes on the Alpine Fault: paleoseismological progress and future directions. *New Zealand Journal of Geology and Geophysics*, 1-20 (2018). https://doi.org:10.1080/00288306.2018.1464658

3 Howarth, J. D. *et al.* Spatiotemporal clustering of great earthquakes on a transform fault controlled by geometry. *Nature Geoscience* (2021). https://doi.org:10.1038/s41561-021-00721-4

4 Hogg, A. G. *et al.* SHCal20 Southern Hemisphere Calibration, 0–55,000 Years cal BP. *Radiocarbon* **62**, 759-778 (2020). https://doi.org:10.1017/RDC.2020.59

5 Ramsey, C. B. & Lee, S. Recent and planned developments of the program OxCal. *Radiocarbon* **55**, 720-730 (2013).

6 Hilton, R. G., Galy, A. & Hovius, N. Riverine particulate organic carbon from an active mountain belt: Importance of landslides. *Global Biogeochemical Cycles* **22** (2008). https://doi.org:https://doi.org/10.1029/2006GB002905

7 Wang, J. *et al.* Long-term patterns of hillslope erosion by earthquake-induced landslides shape mountain landscapes. *Science Advances* **6**, eaaz6446 (2020). https://doi.org:10.1126/sciadv.aaz6446

8 Hilton, R. G. & West, A. J. Mountains, erosion and the carbon cycle. *Nature Reviews Earth & Environment* **1**, 284-299 (2020). https://doi.org:10.1038/s43017-020-0058-6
