# Supplementary material for: Long term carbon export from mountain forests driven by hydroclimate and extreme event driven landsliding
Source: Commun Earth Environ. 2025 Jun 4;6(1):432. doi: 10.1038/s43247-025-02382-2 (PMC12137134; doi:10.1038/s43247-025-02382-2)
Supplement: Supplementary file 3 — description of additional supplementary file [file 43247_2025_2382_MOESM3_ESM.pdf]

Description of Additional Supplementary Files:

File name: Supplementary Material

Description:

PDF of supplementary material for Howarth et al. (in press), including supplementary Figures 1 through 9.

File name: Supplementary Data 1

Description:

Volumes, densities, water contents and sediment and carbon masses for lakes Paringa and Mapourika.

File name: Supplementary Data 2

Description:

Suspended sediment, OC_{bio} and OC_{petro} yields for lakes Paringa and Mapourika.

File name: Supplementary Data 3

Description:

Organic geochemistry measurements for Lake Mapourika.

File name: Supplementary Data 4

Description:

^{137}Cs measurements for master cores from lakes Paringa and

Mapourika.

File name: Supplementary Movies 1

Description:

Movie of reconstructed sedimentary fill volume model for Lake Paringa

File name: Supplementary Movies 2

Description: Movie of reconstructed sedimentary fill volume model for Lake Mapourika
